# NSTEMI treatment: should we always follow the guidelines?

**DOI:** 10.1007/s12471-019-1244-3

**Published:** 2019-03-14

**Authors:** A. W. J. van ’t Hof, E. Badings

**Affiliations:** 1 Department of Cardiology, Maastricht University Medical Centre, Maastricht, The Netherlands; 2 Department of Cardiology, Zuyderland Medical Centre, Heerlen and Sittard, The Netherlands; 3 Department of Cardiology, Isala Hospital, Zwolle, The Netherlands; 4 Department of Cardiology, Deventer Ziekenhuis, Deventer, The Netherlands

Non-ST elevation myocardial infarction (NSTEMI) has been diagnosed more often in recent years, not only since the introduction of high-sensitive troponin assays, but also because of the ageing population. The outcome is rather good in the short term; however, in the long term the outcome is poor due to the high incidence of co-morbidities such as renal failure, diabetes and hypertension. The diagnosis of NSTEMI is rather difficult. During the 2018 ESC meeting an update of the universal definition of MI was presented [1]. A diagnosis of NSTEMI can only be made if acute myocardial injury (defined as a rise and/or fall of cardiac troponin (cTn) above the 99th percentile upper reference limit (URL)) is present in combination with acute myocardial ischaemia. Type 1 MI is caused by atherosclerosis (plaque rupture or erosion), whereas type 2 MI is the result of an imbalance between oxygen demand and supply (hypertension, anaemia or tachycardia) (Fig. 1). Only patients with type I MI might benefit from early angiography and/or revascularisation; however, in daily practice it is not easy to discriminate between type 1 and type 2 MI.

**Fig. 1:**
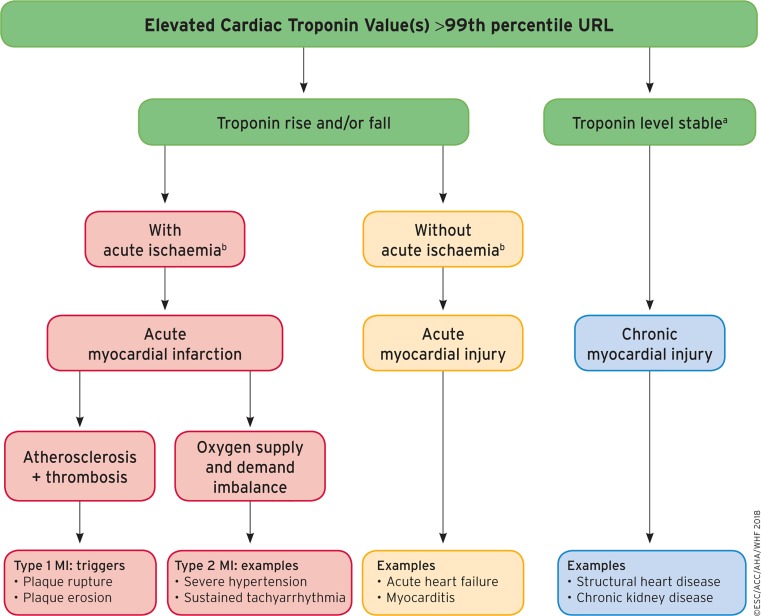
Model for interpreting myocardial injury (^a^Stable denotes <20% variation of troponin values in the appropriate clinical context, ^b^Ischaemia denote signs and/or symptoms of clinical myocardial ischaemia,* MI* myocardial infarction, *URL* upper reference limit). With permission of Oxford Press.

The most recent NSTEMI guidelines recommend same-day transfer for all high-risk patients who present at a non-PCI centre ([2]; Fig. 2). In the Netherlands this might have serious consequences, as most NSTEMI patients present at non-PCI centres.

**Fig. 2:**
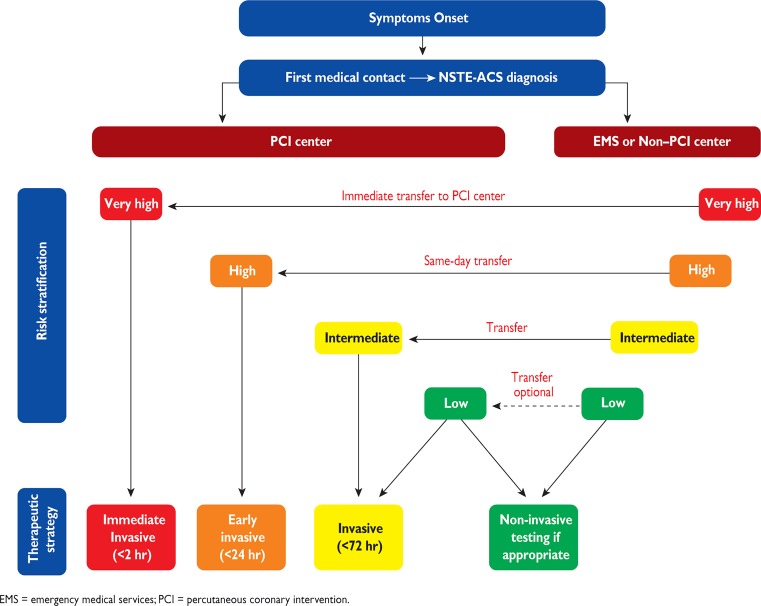
Selection of non-ST-elevation acute coronary syndromes (*NSTEMI-ACS*) treatment strategy and timing according to initial risk stratification (*EMS* emergency medical services, *PCI* percutaneous coronary intervention). (From [2] with permission of Oxford Press)

In this issue of the *Netherlands Heart Journal*, Hoedemaker et al. [3] report the results of a 6-month registry of NSTEMI patients. Almost 900 patients were registered at 23 Dutch non-PCI centres. The first remarkable finding was that almost 15% of patients did not undergo angiography, despite the presence of high (HR) or very high risk (VHR) criteria. Secondly, only 58% of VHR and 61% of HR patients underwent angiography within the time frames (<2 h (VHR) and <24 h (HR)) recommended by the 2015 ESC NSTEMI guideline.

## Same-day transfer

The decision concerning transfer to a PCI centre was at the discretion of the cardiologist at the non-PCI centre. Almost 56% of patients had an indication for same-day transfer because of the presence of at least one HR criterion (most often rise and/or fall in cTn); however, only 25% were transferred to a PCI centre. These transferred patients were younger and more often underwent revascularisation, suggesting a selection of specific patients. Routine same-day transfer of all HR patients might only benefit those with an indication for revascularisation (percutaneous coronary intervention (PCI) or coronary artery bypass grafting (CABG)), but not for those with an indication for medical therapy. Only 43% of patients underwent PCI, whereas 36% were medically treated. This report shows that it is highly questionable whether routine same-day transfer of all high-risk NSTEMI patients to a PCI centre, as recommended by the ESC guideline, is cost-effective. Although the report of Hoedemaker et al. [3] does not provide a definite answer, it suggests that a more selective approach to referral might be non-inferior and perhaps more cost-effective. A serious limitation is the lack of outcome data, and a lack of the reasons why coronary angiography was not performed in 15% of patients. The strength of the report is the description of daily practice in a nationwide registry of non-selected patients. Guidelines are often based upon reports from randomised clinical trials of selected patients and might not be applicable for all patients with suspected coronary ischaemia. The implementation of guidelines is highly dependent on national organisation and logistics. In the Netherlands, many non-PCI centres have facilities for cardiac catheterisation, whereas in many hospitals outside the Netherlands or Europe, many patients present to hospitals where no catheterisation is possible and rapid transfer to a more specialised cardiac centre might be more effective in this kind of situation. When reading the text of the 2015 guideline in detail, it states that high-risk NSTEMI patients should be transferred the same day to hospitals with on-site catheterisation facilities in order that they can undergo invasive coronary angiography within 24 h. This is different from the statement in Fig. 2 of the guideline, in which all HR patients presenting at non-PCI centres should be transferred to a PCI centre. So there is a discrepancy between the text and the figure in the guideline.

Another reason why it is not necessary to transfer all high-risk NSTEMI in the Netherlands is the well-organised regional care with good communication and strict protocols not only with the PCI (heart) centres but also with the emergency ambulance service.

## Time to angiography

Angiography in both VHR and HR patients did not meet the time frames as recommended by the ESC guideline. This might be due to the fact that 24/7 coronary angiography is often not possible at non-PCI centres. This is probably the reason why angiography was performed more rapidly when patients were transferred to PCI centres. Would it be cost-effective to have 24/7 angiography performed at non-PCI centres as well? Recently, Badings et al. [4] reported a post hoc subgroup analysis of the Early or Late Intervention in unStable Angina (ELISA)-3 trial of HR patients randomised at a non-PCI centre to early or late coronary angiography. Although the sample size was small, no difference in infarct size or clinical outcome was present between patients who underwent immediate referral for angiography at the PCI centre (STEMI-like approach) versus a strategy of delayed angiography at the non-PCI centre. In this ELISA trial VHR patients were excluded. It remains undebated that in VHR patients coronary angiography should be performed very early (within 2 h, STEMI-like approach) and the low percentage (58%) of VHR patients who underwent coronary angiography within 2 h in the Dutch registry is therefore a matter of concern.

Patients with chest pain suggestive of cardiac ischaemia who do not show ST elevation have diverse diagnoses, varying between pulmonary embolism, non-cardiac chest pain, takotsubo cardiomyopathy and myocarditis; in the end only a minority are diagnosed as acute MI. A liberal referral strategy of all these patients to PCI centres is unwise. The challenge for the future is the proper selection of patients who might benefit from rapid referral. This might best be done in dedicated departments for emergency cardiac care, and is mostly not dependent on the presence of PCI facilities. The question remains, however, whether further improvement might be achieved if non-PCI centres also perform 24/7 coronary angiography.

The report of Hoedemaker et al. [3] again shows the enormous value of high-quality and nationwide registries on acute MI. Up to now, there is no such registry in the Netherlands. Fortunately the Netherlands Heart Registry (NHR, www.nederlandsehartregistratie.nl) is working on implementing a national registry on acute coronary syndromes during 2019. This might help in further optimising the care for all patients with NSTEMI in the Netherlands.
